# D‑Penicillamine‑Stabilized Gold Nanoclusters as a Selective Fluorescent Sensor for Tetracyclines

**DOI:** 10.1007/s10895-026-04829-x

**Published:** 2026-06-16

**Authors:** Luis Marco-Sabater, Elena Zaballos-García, Jorge Escorihuela, Julia Pérez-Prieto

**Affiliations:** 1 Departamento de Química Orgánica, Facultad de Farmacia y Ciencias de la Alimentación, Universidad de Valencia, Avda. Vicent Andrés Estellés 22, Burjasot, 46100 Valencia Spain; 2 Instituto de Ciencia Molecular (ICMol), Universidad de Valencia, Catedrático José Beltrán 2, Paterna, Valencia Spain

**Keywords:** Nanocluster, Gold, Fluorescence, Sensor, Tetracycline

## Abstract

The development of simple, rapid, and cost‑effective sensing methods for monitoring antibiotic residues in water is crucial due to the widespread use of antibiotics in livestock farming and the consequent risk of environmental contamination. In this study, we present a facile synthesis of gold nanoclusters (AuNCs) stabilized with D‑penicillamine (AuNC@D‑Pen), a thiolated ligand that provides excellent colloidal stability and supports strong photoluminescent properties. The synthetic protocol affords highly uniform nanoclusters whose fluorescence emission, centered at approximately 650 nm, is both intense and stable under typical environmental conditions.The fluorescence of AuNC@D‑Pen undergoes progressive quenching in the presence of tetracycline, enabling a sensitive and reliable optical detection strategy. The detection limit (LOD) was determined to be 2.0 µM (0.9 ppm), with a linear response range extending from 0.5 to 220 µM, which positions this system as a promising tool for practical analytical applications, including routine water‑quality monitoring. In addition, the sensor exhibits excellent selectivity for tetracyclines over common potentially interfering species such as anions, metal ions, and amino acids, highlighting the robustness of the sensing mechanism. Overall, the AuNC‑based probe represents an efficient platform for the rapid, selective, and affordable detection of antibiotic contaminants in aqueous environments.

**Supplementary Information:**

The online version contains supplementary material available at 10.1007/s10895-026-04829-x.

## Introduction

**Scheme 1:**
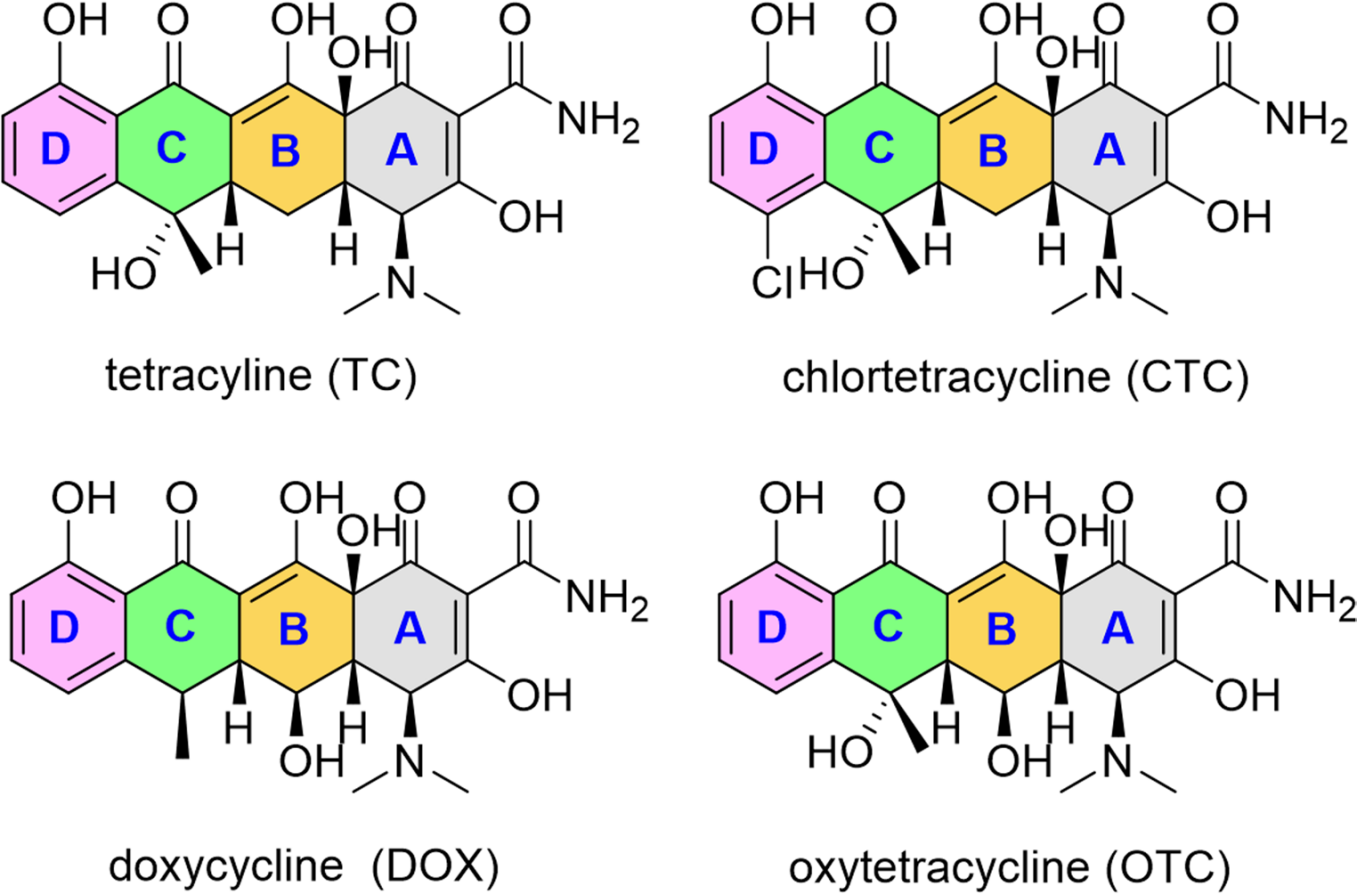
Chemical structure of different tetracyclines

Antibiotics play a critical role in modern healthcare, serving as essential tools for routine clinical treatments, advanced surgical procedures, and the management of infectious diseases worldwide [1, 2]. Among the different families of antibiotics, tetracyclines (TCs) are a group of broad-spectrum antibiotics widely used in veterinary medicine, particularly in poultry, cattle, and swine farming [3]. This family of antibiotics is characterized by a rigid tetracyclic fused nucleus, labeled as A, B, C, and D (Scheme 1), and possesses various functional groups such as hydroxyl, carbonyl, and dimethylamino. Since their discovery in 1948, the extensive use of antibiotics has led to growing concerns over the development of antimicrobial resistance and their accumulation in the environment [4], as large proportions of these antibiotics are excreted unmetabolized and enter aquatic systems [5]. In this regard, tetracycline pollution is considered a global environmental threat due to its extensive use in aquaculture, livestock, and human medicine [6]. Large fractions are excreted unmetabolized (up to 70–90%), subsequently entering soils and aquatic environments, where they are highly persistent because of their hydrophilic nature and resistance to natural degradation [7]. Studies across Europe have detected tetracycline concentrations in surface waters around 0.045 nM (0.02 ppb), but can reach higher concentrations up to 1.22 nM (0.54 ppb) in municipal wastewater treatment plant effluents [8].

The growing concern over antibiotic residues and resistance highlights the need for precise analytical monitoring. In this regard, several analytical methods are used to detect tetracyclines [9], being chromatographic methods, such as HPLC or LC–MS, the most widely employed because of their high precision and sensitivity; however they require expensive instrumentation, long analysis times, and specialized operation, limiting their routine use [10, 11]. On the other hand, electrochemical methods, including those modified with nanomaterials, provide fast response, high sensitivity, and low cost, making them suitable for on‑site analysis, but they generally suffer from matrix interferences, and limited long‑term stability [12, 13]. Electrophoresis methods suffer from limited separation capability and require strict pH control, which hampers their practical applicability [14, 15]. Immunoassay‑based techniques offer high selectivity through antibody recognition, yet they often present higher detection limits and longer assay times compared with emerging sensor technologies [16–18]. Chemiluminescence techniques typically respond to a broad range of compounds, making them suitable only for high-purity samples like pharmaceutical formulations [19, 20]. In contrast, fluorescence analysis has gained significant attention in recent years due to its advantages: low cost, ease of operation, high sensitivity and stability, rapid signal response, real-time detection capability, excellent reproducibility, and minimal sample damage [21]. These attributes make fluorescence-based methods highly promising for the selective and accurate detection of TCs.

In recent years, a wide range of fluorescent nanomaterials, such as quantum dots, carbon-based nanomaterials, rare earth-doped nanoparticles, and metallic nanoclusters, have received growing attention in fluorescence sensing [22]. Among them, metallic nanoclusters (NCs) have emerged as particularly promising nanomaterials due to their exceptional properties, including high photostability, strong photoluminescence, large Stokes shifts, low toxicity, high quantum yields, excellent water solubility, and biocompatibility [23]. Over the past decade, considerable attention has been directed toward the synthesis of silver (AgNCs) and gold nanoclusters (AuNC), which have been widely used as luminescent probes in numerous interdisciplinary applications. Among the various types of metal nanoclusters, gold, silver and copper nanoclusters have been the most extensively investigated [24–26].

AuNC have been stablished as highly effective fluorescent probes for the detection of tetracyclines due to their unique optical properties and strong interactions with antibiotic molecules [27]. These ultrasmall clusters exhibit size‑dependent fluorescence that can be selectively quenched or enhanced when tetracyclines interact with the nanomaterial, enabling sensitive, rapid, and label‑free detection. Their excellent biocompatibility, high quantum yield, and tunable surface chemistry make gold nanoclusters particularly suitable for applications in food‑safety monitoring and environmental analysis, where low detection limits and reliable performance in complex matrices are essential [28]. Along the last decade, different fluorescent AuNC-based sensors have been reported for the detection of TCs. Among them, AuNCs capped with thiolated ligands, such as glutathione [29] and N-acetyl-L-cysteine [30], have shown limits of detection (LOD) of 5.4 and 1.8 µM, respectively (2.4 and 0.8 ppm, respectively). Lower LODs can be achieved using rare-earth metals such as Eu(III) salts. In this regard, systems involving Eu(III) complexes of L-histidine-caped AuNCs [31] or BSA‑stabilized AuNCs [32] allowed the detection of TC with a detection limit of 4.5 nM (2 ppb) in both systems. More complex systems using microfluidic chip with ovalbumin‑stabilized AuNCs in a have achieved the detection of TC in chicken muscle with a LOD of 0.20 µM (90 ppb) [33].

In this study, we describe the preparation of D-penicillamine-capped AuNC (AuNC@D-Pen) via a simple protocol which avoids the use of additional chemicals and complicated synthetic and laborious purification steps. The synthesized AuNC@D-Pen demonstrated strong sensitivity for detecting TCs at low concentrations. Specifically, the characteristic emission at 652 nm showed a clear reduction in intensity upon increasing concentrations of TC, demonstrating strong sensitivity even at low analyte levels. This quenching behaviour is attributed to intermolecular interactions between TC and the ligand‑protected nanocluster surface. The detection method for TCs has the advantages of high sensitivity and selectivity towards other interfering analytes, simple operation, and applicability to environmental water samples.

## Results and Discussion

### Synthesis and Characterization of AuNC@D-Pen

**Fig. 1:**
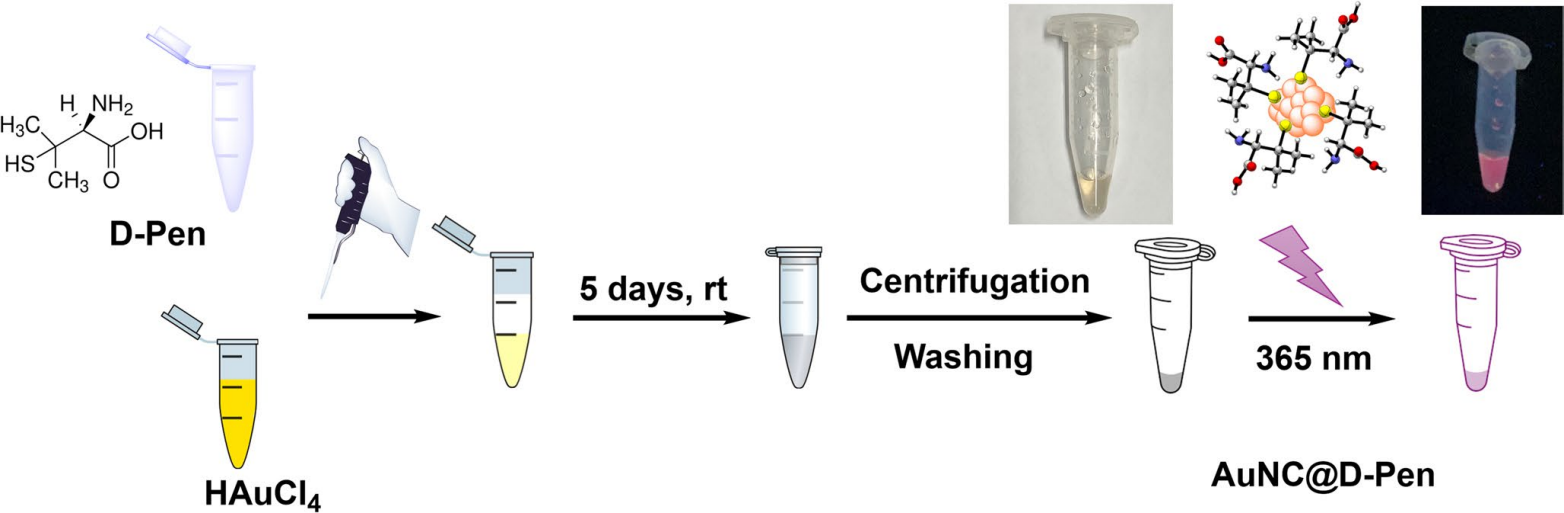
Schematic representation of the synthesis of AuNC@D-Pen

For the preparation of AuNC@D-Pen, we used the bottom-up approach that involves assembling atoms or molecules into nanoclusters with atomic precision [34]. The advantage of this method is that it offers excellent control over size, composition, and surface chemistry, making it ideal for producing atomically precise gold nanoclusters, often with sizes around 2 nm. To this purpose, a freshly prepared 1 mL of an aqueous solution of HAuCl_4_ (50 µL, 50 mM) and another aqueous solution of D-penicillamine (53 µL, 1 M). Both solutions were mixed in a 1.5 mL Eppendorf tube. After a few days, we monitored the formation of gold nanoclusters by checking their fluorescence with a UV lamp (365 nm). After 5 days, a white precipitate appeared and was isolated by centrifugation, washed with water, and isolated, again, by centrifugation. The centrifugation and washing cycles were repeated until no detectable free ligand remained in the supernatant. After purification, the water suspension was stored at room temperature. A schematic representation of the synthesis is shown in Fig. 1.

The UV–Vis spectra of D‑penicillamine exhibits a maximum absorption peak in the UV region at 214 nm, typically associated with n→σ* and n→π* transitions of the sulfhydryl and amine groups. In contrast, HAuCl₄ displays The absorption bands at 292 and 216 nm are attributed to ligand-to-metal charge transfer transitions [35]. The synthesized AuNC@D‑Pen showed an absorption band at 310–320 nm, whereas the D-penicillamine ligand does not show any such absorption feature in that range (Fig. S1). Furthermore, he synthesized AuNCs did not exhibit any peak around 520 nm in the UV spectrum, indicating the absence of the SPR band characteristic of AuNPs.

**Fig. 2:**
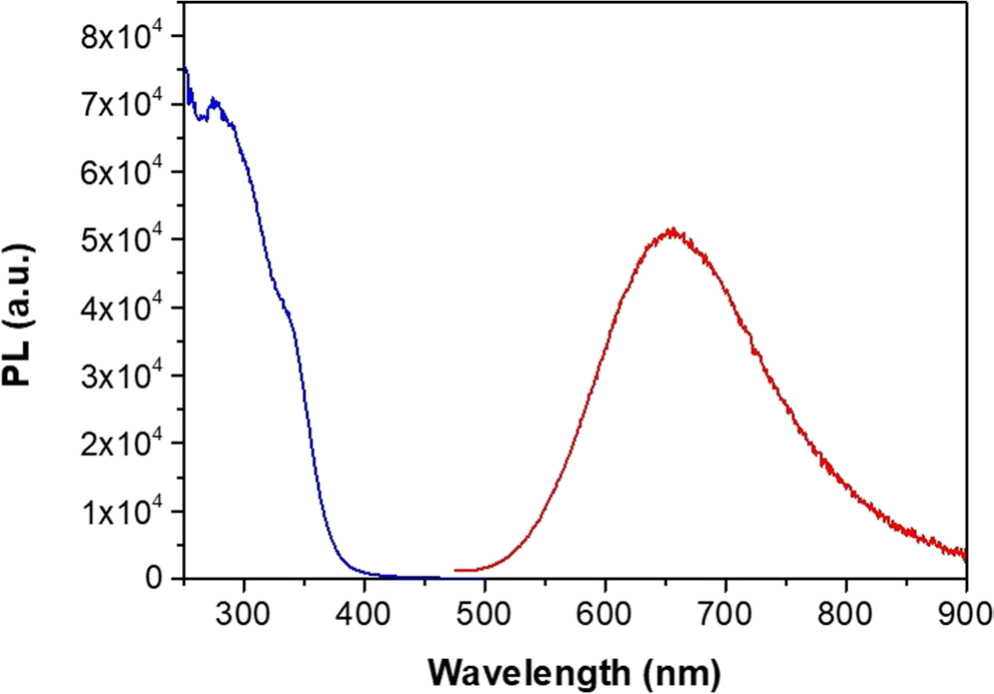
(**a**) UV-Vis spectra of HAuCl_4_ solution in water. (**b**) Emission (**red**) and excitation (**blue**) spectra of AuNC@D-Pen

The photoluminescence properties of AuNC@D‑Pen were investigated by recording their emission spectrum upon excitation at 310 nm. As shown in Fig. 2b (red line), the nanoclusters exhibited a broad emission band with an emission maximum centred at 652 nm. This wide spectral profile is characteristic of ligand‑protected gold nanoclusters, whose electronic transitions arise from discrete energy levels rather than the band‑like structure typical of larger nanoparticles. Importantly, the emission spectrum remained unchanged when the excitation wavelength varied between 300 and 420 nm, demonstrating that the luminescence originates from intrinsic, relaxed excited states of the nanoclusters. The absence of excitation‑dependent shifts indicates that the observed signal is true photoluminescence rather than an artefact from scattering, surface defects, or heterogeneous emissive species.

The AuNC@D‑Pen sample displayed a photoluminescence quantum yield (φₚₗ) of 1%. Although modest, this quantum yield reflects efficient relaxation pathways within the metal–ligand framework and confirms that D‑penicillamine provides a stable surface environment supporting radiative recombination. This value is consistent with previously reported ligand-protected gold nanoclusters, particularly those stabilized by thiolated amino acids or small chiral ligands, which typically exhibit quantum yields in the sub‑percent to few‑percent range [36].

The molar ratio of gold: ligand is a critical parameter in the bottom-up synthesis of gold nanoclusters, as it strongly influences the size, monodispersity, stability, and surface chemistry of the nanoclusters [37]. The molar ratio was investigated for D-penicillamine to obtain high quality luminescent gold nanoclusters. Under the described conditions, the concentration of HAuCl_4_ was kept constant and different HAuCl_4_/D-Pen molar ratios were assayed: 1:1, 1:2, 1:5, 1:10, 1:20. After recording to the fluorescent spectrum of the nanocluster under different molar ratios, the highest fluorescence intensity gradually decreased with the HAuCl_4_/D-Pen molar ratio with a maximum of intensity for the nanocluster with the 1:20 HAuCl_4_/D-Pen molar ratio (Fig. S2). Based on these observations, we conclude that the optimal molar ratio for synthesizing strongly luminescent AuNC@D‑Pen is 1:20, ensuring both efficient surface stabilization and maximized fluorescence.

The influence of reaction time using a HAuCl_4_/D-Pen molar ratio of 1:20. was also investigated. A progressive increase in the fluorescence intensity was observed during the first several days of incubation, indicating ongoing structural formation of the AuNC@D‑Pen system. The fluorescence reached its maximum after approximately five days (Fig. S3), suggesting that this period is required for the nanoclusters to fully develop their optimal luminescent properties.

The ^1^H NMR spectrum of D‑penicillamine displayed two characteristic peaks at 1.41 and 1.49 ppm, corresponding to the protons of the methyl groups, along with a signal at 3.62 ppm assigned to the chiral methine (CH) proton. Upon coordination to gold and formation of AuNC@D‑Pen, both methyl peaks experience a slight downfield shift to 1.43 and 1.51 ppm, respectively, while the methine proton shifts to 3.82 ppm (Fig. S4). These changes in chemical shift are indicative of ligand–metal interactions and support the successful incorporation of D‑penicillamine onto the AuNC surface.

**Fig. 3:**
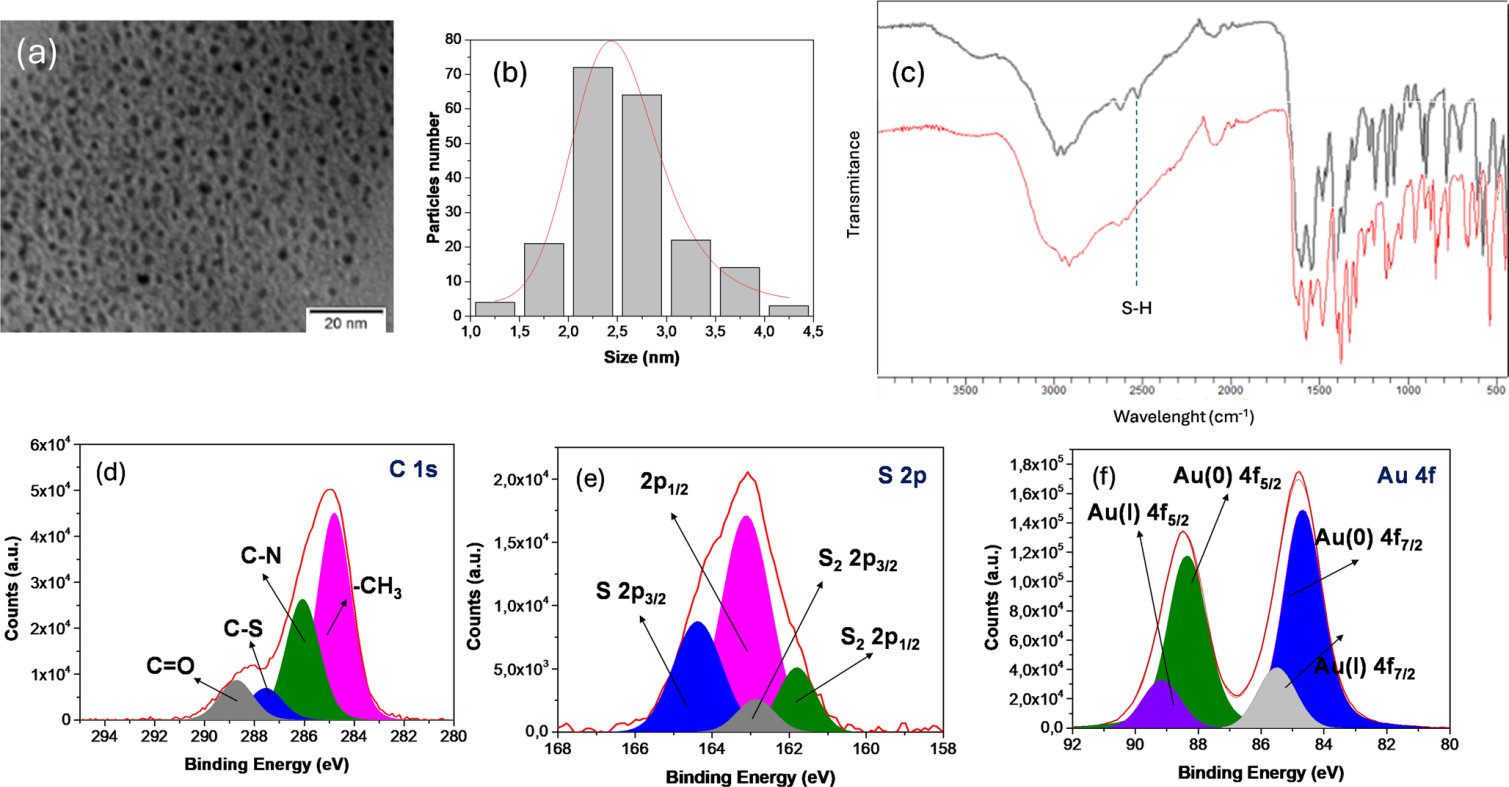
Physicochemical characterization (**a**) TEM image of AuNC@D-Pen. (**b**) Representative histogram of particle size. (**c**) FTIR spectra of D-penicillamine (**black line**) and AuNC@D-Pen (**red line**). XPS high-resolution spectra of (**d**) C 1s, (**e**) S2p and (**f**) Au 4f of AuNC@D-Pen

The morphology of the AuNC@D-Pen was characterized using transmission electron microscopy (TEM). As shown in Fig. 3a, the micrographs of the prepared AuNC@D-Pen exhibited uniform dispersion and predominantly spherical morphology. Importantly, the particles appear well isolated from one another, with no evidence of large agglomerates or significant clustering. This lack of aggregation indicates that D‑penicillamine provides effective surface stabilization, preventing particle–particle fusion and maintaining colloidal stability during synthesis and imaging. Detailed quantitative analysis of the TEM images further confirms the presence of spherical gold nanoclusters with an average diameter of 2.5 ± 0.4 nm, a size range characteristic of well‑defined, ligand‑protected Au nanoclusters. This particle size is consistent with previously reported penicillamine-capped AuNCs (Fig. 3b**)** [38–40]. This narrow size distribution is consistent with controlled nucleation and growth processes typically observed in thiolate‑stabilized gold nanocluster systems, supporting the successful formation of small, monodisperse AuNCs.

The functional groups on the surface of AuNC@D-Pen were characterized by Fourier Transform Infrared (FTIR) spectroscopy. FTIR spectroscopy is a powerful tool for characterizing gold nanoclusters, especially when they are functionalized with thiolated ligands [41]. D-penicillamine exhibits the S–H stretching band in the region of 2550–2600 cm⁻¹, and upon binding to gold, this band disappears or is significantly reduced, indicating formation of Au–S bonds. As shown in Fig. 3c, the band corresponding to the S-H bond in D-penicillamine disappears when the ligand coordinates gold atoms. The loss of the S–H band therefore confirms ligand and highlights the high affinity of sulfur for gold, a well‑known feature that explains the stability of thiolate‑protected gold nanoclusters.

X-ray photoelectron spectroscopy (XPS) measurements were performed to investigate the electronic structure and surface chemical composition of the gold nanoclusters. The XPS analysis provides insights into the oxidation states of gold and the nature of the chemical interactions between gold atoms and stabilizing ligands [42]. In particular, analysis of the C 1s core-level spectra showed multiple components (Fig. 3d), typically associated with different carbon bonding environments such as C–C/C–H (~ 284.8 eV), C–N/C–O (~ 286.2 eV), C–S (~ 287.4 eV)and O–C = O (~ 288.5 eV), which are indicative of the different carbon environments present in penicillamine ligand used to stabilize the AuNCs.

The high-resolution S 2p spectra provides information of the bonding environment of sulphur atoms originating from thiol-based ligands. The S 2p region exhibited characteristic doublet peaks, corresponding to the spin-orbit components S 2p₃/₂ and S 2p₁/₂, typically observed around 162–164 eV (Fig. 3e). Peaks near ~ 162 eV are indicative of sulphur atoms covalently bonded to gold (Au–S), confirming thiolate-gold interactions [43, 44].

Finally, the Au 4f spectra is generally used to confirm the metallic state of gold within the clusters. As shown in Fig. 3f, the high-resolution Au 4f spectra revealed characteristic doublets corresponding to Au^0^ and the oxidized species (e.g., Au⁺), as inferred from the peaks at 84.7 and 88.4 eV for Au^0^, and 85.3 and 89.0 eV for Au^+^ [44–47].

Next, we evaluated the stability of AuNC@D-Pen at different pH values (Fig. S5). The stability of gold nanoclusters is highly dependent on the pH of the surrounding environment, as pH can influence their surface charge, ligand conformation, aggregation behaviour, among other effects [48]. For these experiments, were prepared AuNC@D-Pen solutions with similar value for the absorbance at 310 nm around, and then small amounts of a stock solution of NaOH 0.1 M was added to adjust the pH to the desired value. After shaking and allowing the sample resting at room temperature for 5 min, the fluorescence at 650 nm was measured following excitation at 305 nm. The results indicate that the fluorescence intensity of AuNC@D-Pen at 650 nm was kept constant and near the maximum value in the pH range from 2.1 until 4.9. However, at pH = 6.2 the fluorescence decreased dramatically until pH = 8.9. This effect may be attributed to the degree of deprotonation of the organic ligand with the pH. This behaviour was irreversible as when reaching pH = 8.9, we added acid (HCl 0.5 M) until pH = 3.8, and the original value of fluorescence was not recovered [49].

### AuNC@D-Pen for Fluorimetric Detection of Tetracycline

**Fig. 4:**
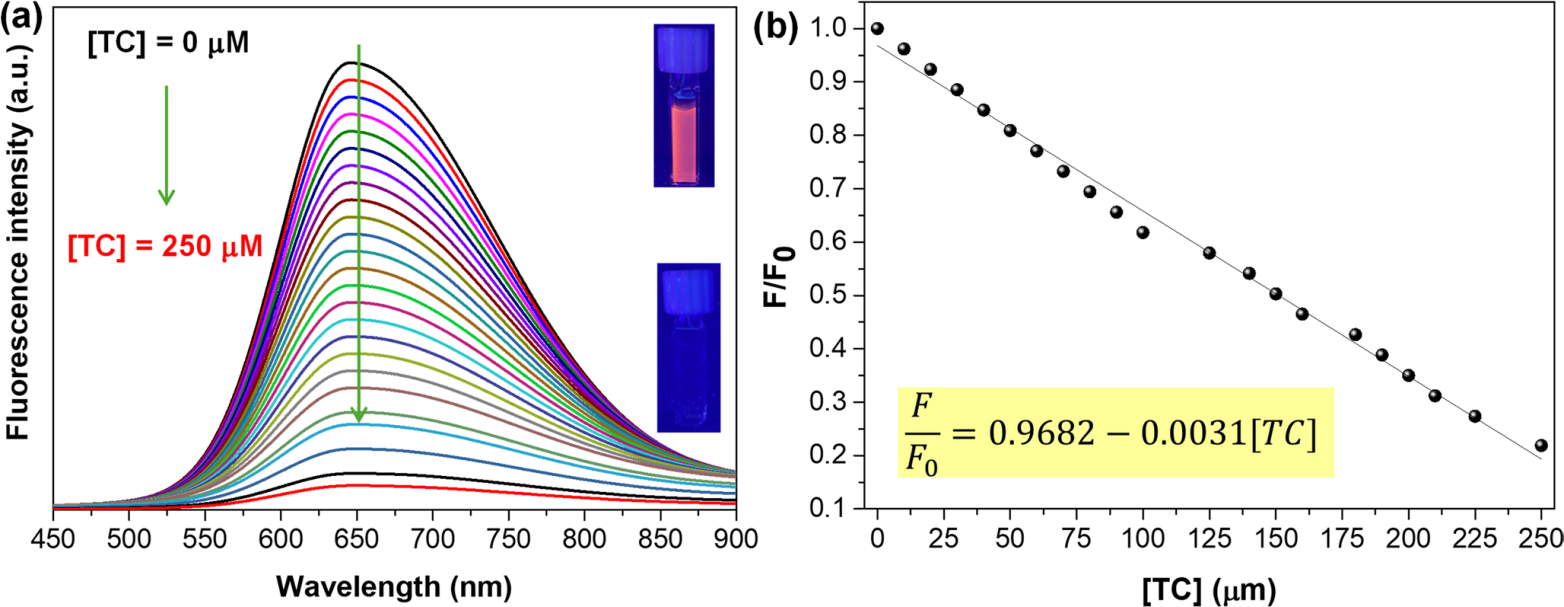
(**a**) PL spectra of AuNC@D-Pen upon addition of [TC] from 0 to 250 µM. (**b**) Relationship between the relative PL intensity F/F_0_ and the concentration of TC in the range 0.5–250 µM, where F_0_ represents the PL intensity in the absence of TC and F represents the PL upon addition of TC

Under the optimal experimental conditions described previously, we evaluated the analytical performance and sensitivity of the ratiometric fluorescence sensor for tetracycline (TC) detection. As shown in Fig. 4a, the fluorescence intensity of AuNC@D-Pen at 652 nm progressively decreased as the concentration of TC increased. Because of this well-defined fluorescence response, the intensity ratio F/F_0_ exhibited a clear and proportional dependence on TC concentration. When fitting the data, a strong linear correlation was obtained for concentrations ranging from 0.5 to 220 µM (Fig. 4b). The calibration curve followed the equation: F/F_0_ = 0.9682 − 0.0031 [TC]/µM, with an excellent correlation coefficient (R² = 0.99344), demonstrating high analytical reliability. Based on the 3σ criterion, the limit of detection (LOD) was determined to be 2.0 µM (0.9 ppm), highlighting the good sensitivity of the sensing nanomaterial.

**Fig. 5:**
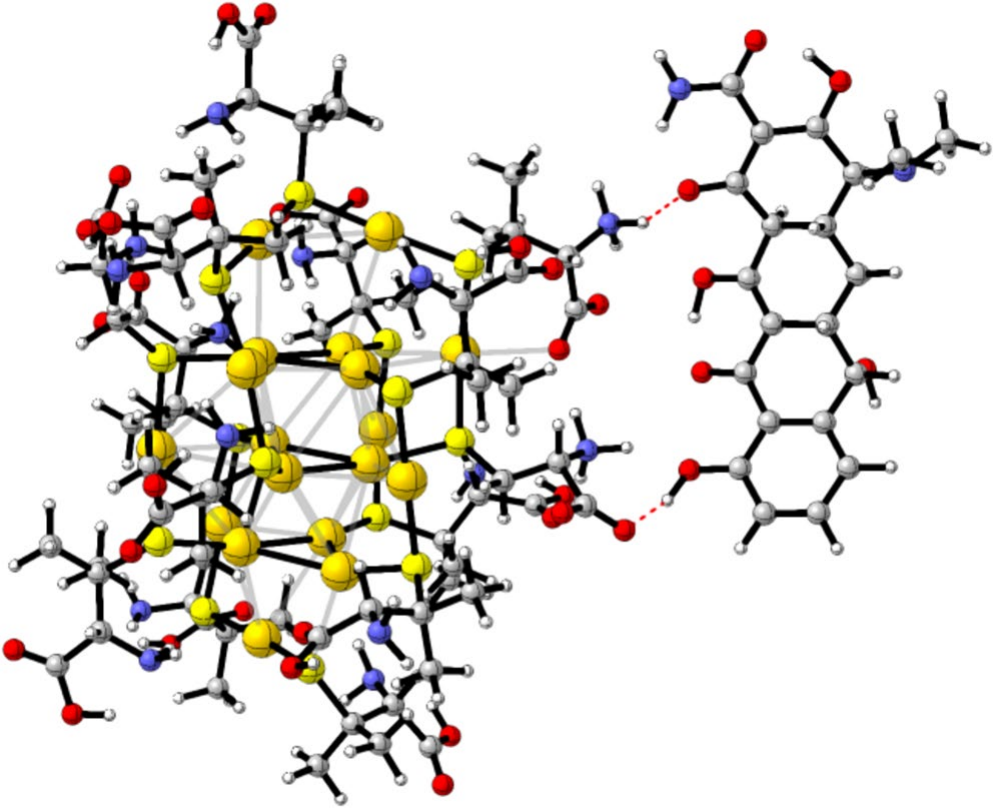
Plausible interaction between AuNC@D-Pen and TC

We believe that the interaction between TC and AuNC@D-Pen might involve intermolecular hydrogen bonds between the hydroxyl (–OH) and carbonyl (C = O) groups located on one of the aromatic rings of the tetracycline scaffold as illustrated in Fig. 5. To support this hypothesis, we performed density functional theory (DFT) calculations using Gaussian 16 [50] at the B3LYP‑D3/def2‑TZVP level of theory [51, 52], employing the SDD pseudopotential for gold [53]. This model was chosen to capture key electronic interactions and hydrogen-bonding trends, rather than exact structural replication. To this end, we used a model consisting of 18 gold atoms and 14 D-penicillamine ligands. The optimized structure obtained from the computational study reveals the formation of hydrogen bonds between tetracycline’s OH and C = O groups and the functional groups of the D-penicillamine ligand shell. These results support the proposed interaction model and align well with previously reported systems, where hydrogen bonding plays a major role in adsorption, orientation, and stabilization [30].

Sensor selectivity is a critical parameter, particularly when detecting specific analytes within complex matrices. High selectivity ensures that the sensor responds primarily to the target compound, minimizing interference from other substances that may be present in the sample [54]. This is especially important in real-world applications, such as food safety, environmental monitoring, or clinical diagnostics, where samples often contain a multitude of species which can interact increasing or decreasing the signal.

**Fig. 6:**
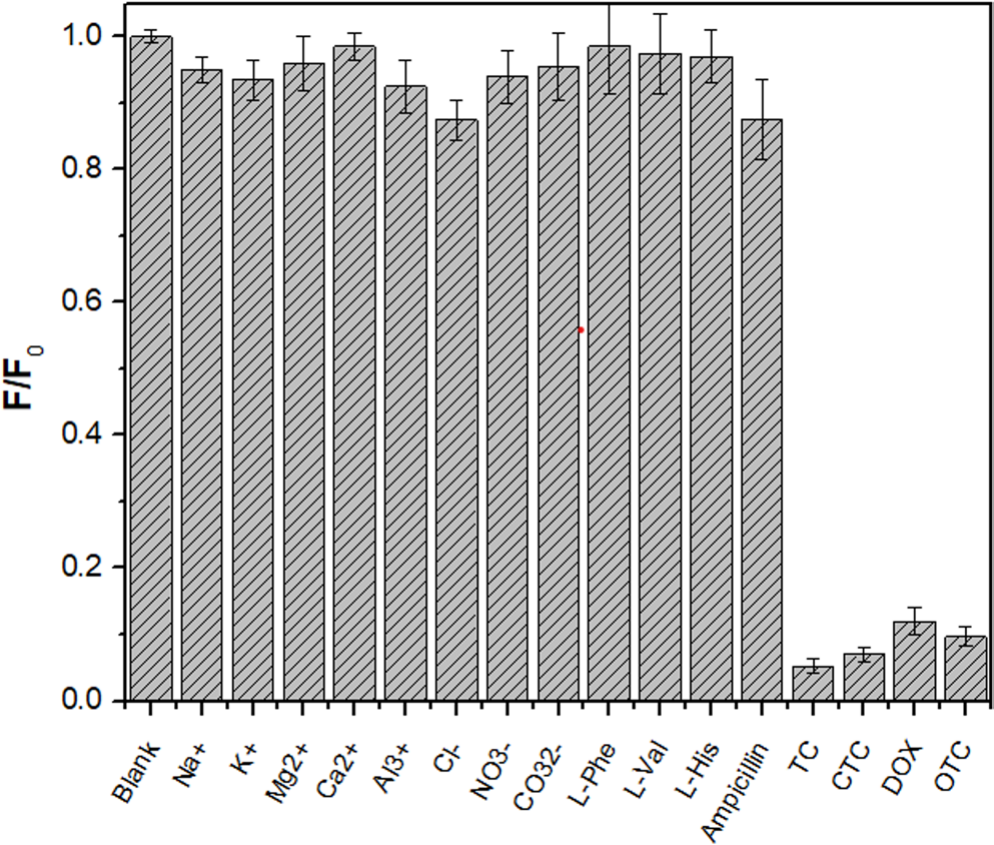
Selectivity of AuNC@D-Pen against other compounds

Thus, we evaluated the selectivity of the AuNC@D-Pen sensor towards tetracycline by exposing the nanomaterial to a selection of potentially interfering ions and molecules. For this purpose, we prepared stock solutions containing cations such as Na^+^, K^+^, Mg^2+^, Ca^2+^, Al^3+^, anions such as Cl^−^, NO_3_^−^, CO_3_^2−^; natural amino acids such as phenylalanine (L-Phe), valine (L-Val), or histidine (L-His), another antibiotic, ampiciline, and other structurally related TC analogues such as CTC, DOX or OTC. All solutions were acidified until pH = 4. The fluorescence intensity response of AuNC@D-Pen to each interfering analyte was measured at an excitation wavelength of 310 nm. As shown in Fig. 6, the fluorescence intensity ratio remained almost unchanged (a slight decrease was observed in some cases) in the presence of these interfering species, indicating minimal non-specific interactions. These potential interferents have a negligible impact on the fluorescence signal, which remains largely unaffected in their presence. In contrast, the addition of a solution of TC or other structurally related TC analogues induced a quenching in the fluorescence, confirming the sensor’s strong and specific interaction with TCs. The sensor can effectively detect different tetracyclines because, according to the proposed DFT model, the interaction between TC and the penicillamine ligand occurs at a common binding region shared by all TCs under study. These results clearly demonstrate that the AuNC@D-Pen sensor possesses high selectivity for TCs over other coexisting substances, making it a reliable and robust platform for the selective detection of tetracycline in complex sample environments.

The long‑term stability of AuNC@D‑Pen is a critical parameter for assessing the practical applicability of the proposed sensing platform. Therefore, we evaluated the long‑term storage stability of AuNC@D‑Pen under typical laboratory conditions. The AuNC@D‑Pen aqueous dispersions were stored at 4 °C in the dark, and their fluorescence intensity was periodically monitored over time. The results showed that the fluorescence signal remained largely stable, retaining approximately 90% of its initial intensity after 12 weeks of storage, with no noticeable changes in emission peak position or spectral shape. This indicates good photophysical stability and minimal aggregation or degradation during storage, making it suitable for routine laboratory use and short‑term deployment.

### Tetracycline Detection in Real Samples

To evaluate the practical applicability of the proposed method for tetracycline detection in real samples, we conducted assays in tap water and lake water. To this end, different amounts of TCs (0.8, 1.2, 2.5 and 5.0 µM) were added into water samples, the pH was adjusted to 3.0, analysed using the obtained calibration curve and recovery tests in three repeated measurements were performed and the relative standard deviation (RSD) was obtained. As shown in Table 1, the recovery values ranged from 96.2% to 104.4%, demonstrating excellent accuracy and minimal matrix interference. The low RSD values further confirm the good repeatability of the measurements. These results indicate that the AuNC@D-Pen sensor possesses acceptable results for TC determination in a water samples.

**Table 1:** TC determination in real samples

Sample	Spiked (µM)	Detected ± SD	RSD (%)	Recovery (%)
Tap water	0.8	0.82 ± 0.05	6.1	102.5
	1.2	1.24 ± 0.09	7.3	103.3
	2.5	2.43 ± 0.19	7.8	97.2
	5.0	5.14 ± 0.11	2.1	102.8
Lake water	0.8	0.77 ± 0.06	7.8	96.3
	1.2	1.25 ± 0.11	8.8	104.2
	2.5	2.61 ± 0.21	8.0	104.4
	5.0	5.21 ± 0.22	4.2	104.2

## Conclusions

In conclusion, the findings presented in this work demonstrate the development of a rapid and moderately sensitive assay for detecting tetracycline, based on the fluorescence quenching of gold nanoclusters stabilized with D-penicillamine. The AuNC@D-Pen system displayed a fluorescence peak at 652 nm, attributed to the intrinsic emission of D-Pen-stabilized AuNCs. Upon the addition of tetracycline, the fluorescence intensity at 652 was quenched, offering a linear detection range from 0.5 to 220 µM and a low LOD of 2.0 µM. The sensor was successfully validated in real water samples, demonstrating its practical applicability. The AuNC@D-Pen system offers distinct advantages, including straightforward preparation, rapid response time, broad linear range, good sensitivity and selectivity. This work may inspire the development of additional fluorescence-based antibiotic sensors utilizing gold nanoclusters. Based on these findings, ongoing research in our laboratory is focused on engineering other nanostructures that combine AuNCs with thiolated ligands to enhance sensitivity and broaden the detection spectrum.

## Supplementary Information

Below is the link to the electronic supplementary material.

Supplementary Material 1 (DOCX 214 KB)

## Data Availability

No datasets were generated or analysed during the current study.
