# Supplementary material for: D‑Penicillamine‑Stabilized Gold Nanoclusters as a Selective Fluorescent Sensor for Tetracyclines
Source: J Fluoresc. 2026 Jun 16;36(6):4029–37. doi: 10.1007/s10895-026-04829-x (PMC13331949; doi:10.1007/s10895-026-04829-x)
Supplement: Supplementary file 1 — Supplementary Material 1 (DOCX 214 KB) [file 10895_2026_4829_MOESM1_ESM.docx]

*Supporting Information*

D‑Penicillamine‑Stabilized Gold Nanoclusters as a Selective Fluorescent Sensor for Tetracyclines

Luis Marco-Sabater, Elena Zaballos-García, Jorge Escorihuela* and Julia Pérez-Prieto*

# **1. Experimental details.**

## **1.1. Materials.**

All the chemicals and solvents used in this thesis degree were of analytical grade and used without any additional purification. Gold(III) chloride (HAuCl_4_·3H_2_O), sodium chloride (NaCl), potassium chloride (KCl), magnesium chloride (MgCl_2_), calcium chloride (CaCl_2_), aluminum chloride (AlCl_3_), sodium nitrate (NaNO_3_), sodium carbonate (Na_2_CO_3_), copper sulphate (CuSO_4_), L-phenylalanine (L-Phe), L-valine (L-Val), L-histidine (L-His), tetracyclines and ampicillin were purchased from Sigma-Aldrich. D-penicillamine was purchased from BLD Pharmatech. For all aqueous solutions, high purity deionized water from a Millipore system was used*.*

## **1.2. Equipment.**

The following technical instruments were used for analysis and characterization. Centrifugation was performed on a Beckman Coulter's Microfuge 16 benchtop centrifuge. UV-vis absorption spectra were recorded on a PerkinElmer 1050+ UV/vis/NIR spectrometer. All the measurements were performed using 1cm×1cm path length quartz cuvettes. Fluorescence spectra were recorded on a FLS1000 photoluminescence spectrometer from Edinburgh Instruments. The quantum yield was measured with a Hamamatsu C9920-02 absolute PL Quantum Yield Measurement System. The pH measurements were carried out by using a Crison GLP 21 pH meter. Transmission electron microscopy (TEM) images were acquired using a HITACHI HT7800 microscope with a filament of LaB6 operating at 100 kV. Fourier transform infrared (FT-IR) spectra were recorded on a Thermo Scientific Nicolet iS10. X-ray photoelectron spectroscopy (XPS) spectra were acquired with VG-Microtech Multilab 3000 equipment. The ^1^H spectrum were registered at room temperature in a Bruker AvanceIII 300 spectrometer, with a 300 MHz Bruker magnet. The chemical shifts (δ) are reported in ppm using deuterium oxide, 99.9% atom (D_2_O) as solvent.

## **1.3. Procedures.**

### **1.3.1. Preparation of AuNC@D-Pen.**

A freshly prepared aqueous solution of HAuCl_4_ (50 µL, 50 mM) was diluted in water (1 mL), and an aqueous solution of D-penicillamine (53µL, 1 M) was added. The mixture was set for 5 days at room temperature, obtaining a white precipitated (fluorescent under UV-light) and a colourless solution. The precipitate was isolated by centrifugation at 10000 rev/min for 15 min. The supernatant was slowly removed without disturbing the precipitate, which had been washed two times by dispersion in water and precipitation by centrifugation at 10000 rev/min for 15min. The centrifugation and washing cycles were repeated until no detectable free ligand remained in the supernatant. After purification, the **AuNC@D-Pen** were dispersed in water.

### **1.3.2. Fluorescence detection of tetracycline.**

Fluorescence experiments were conducted at room temperature using an excitation wavelength of 310 nm. TC solutions of varying concentrations were freshly prepared and sequentially added to 3 mL of **AuNC@D-Pen** solution (pH 4.3, with an absorbance of 0.4 at 305 nm). Photoluminescence spectra were recorded at room temperature immediately after each addition of tetracycline. The concentration of tetracycline was plotted on the x-axis, while the corresponding PL intensity was plotted on the y-axis. Finally, a linear correlation curve was generated to determine the concentration of tetracycline.

### **1.3.3. Selectivity studies of AuNC@D-Pen.**

To evaluate the selectivity of the prepared **AuNC@D-Pen**, a series of selective experiments were performed. Initially, 5 μL of a 0.05 M TC solution was added to a solution of **AuNC@D-Pen** to establish the baseline photoluminescence (PL) intensity as a control. Subsequently, 5 μL of 0.05 M solutions of different potential interfering analytes, such as common anions and cations (NaCl, KCl, MgCl_2_, AlCl_3_, Na_2_CO_3_, CaCl_2_, and MgSO_4_), amino acids (L-Phe, L-Val, and L-His), and ampicillin, were added to the AuNC@D-Pen solution. Fluorescence intensity values were measured after an incubation time of 5 minutes at room temperature.

### **1.3.4. Selectivity studies of AuNC@D-Pen.**

We performed recovery experiments using both tap water and lake water. Tap water was tested for TC without pretreatment. For TC detection in lake water, the sample was initially centrifuged to remove suspended particles. Next, water samples were adjusted at pH around 4.0 by adding HCl solution. Known concentrations of TC (0.8, 1.2, 2.5, and 5.0 µM) were added to each water sample, and the resulting solutions were analysed under the same optimized conditions used for the calibration curve. For each concentration level, three independent measurements were carried out, allowing the calculation of both the recovery percentage and the corresponding relative standard deviation (RSD).

## **1.4 Additional figures.**


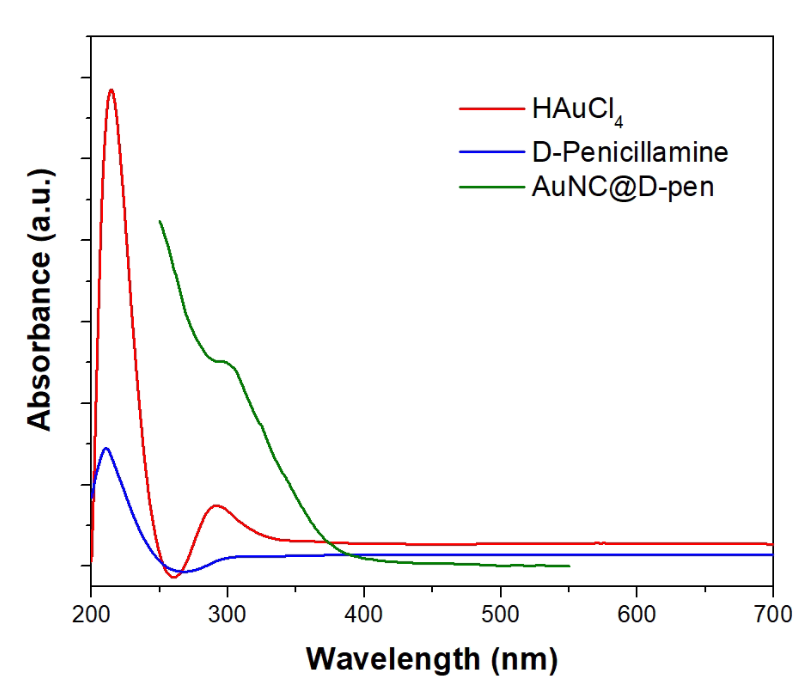


**Figure S1.** UV-Vis spectra of HAuCl_4_, D-Penicillamine AuNC@D-Pen in water.

**Figure S2.** HAuCl_4_/D-Pen molar ratio effect on the fluorescence of AuNC@D-Pen.


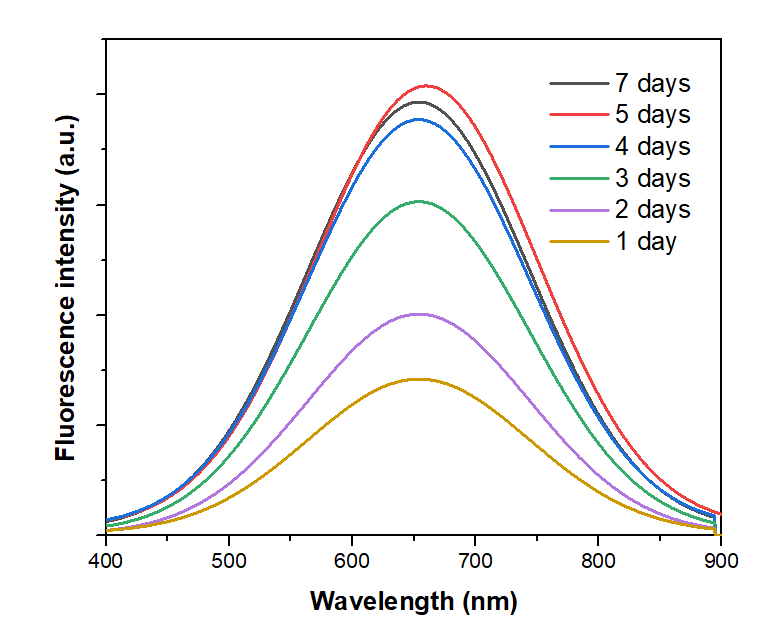


**Figure S3.** Reaction time effect on the fluorescence intensity of AuNC@D-Pen.

##

**Figure S4.** ^1^H NMR (300 Mz, D_2_O) of D-Penicillamine (top) and D-Penicillamine and HAuCl_4_ (**AuNC@D-Pen**) (bottom).


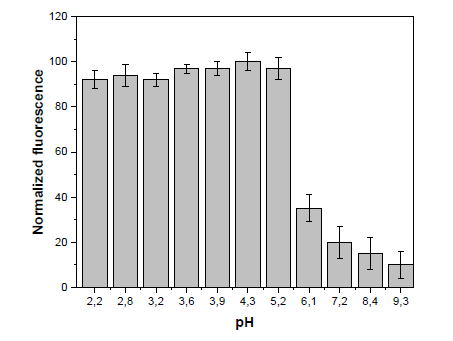


**Figure S5.** pH effect on the fluorescence intensity of **AuNC@D-Pen**.

## **1.5. Computational details.**

DFT calculations were performed at the B3LYP-D3/def2TZVP (SDD for Au) level of theory using Gaussian 16. To this end, a model consisting of 18 gold atoms and 14 D-penicillamine ligands was modeled.

### **1.5.1. Cartesian coordinates of optimized structure**

79 0.241805000 -0.673956000 1.653161000

79 -2.257242000 0.928007000 0.383311000

79 0.691109000 1.641077000 0.107229000

79 1.100526000 -1.004837000 -1.055552000

79 -2.986748000 -1.780430000 2.528374000

79 -1.749724000 -1.647886000 -0.531926000

79 -1.198754000 0.578617000 -2.167480000

79 -0.604419000 2.634307000 2.807478000

79 0.601400000 -3.997344000 0.782606000

79 -1.986206000 3.537804000 -1.542843000

79 2.909327000 -1.607299000 1.075347000

79 3.413898000 0.671608000 -0.439024000

79 1.896376000 1.912711000 -3.318935000

79 4.363176000 -1.869320000 -2.112081000

79 2.863827000 1.069737000 2.438293000

79 -4.818733000 -1.916877000 -0.812376000

79 -4.471415000 1.002174000 -2.713708000

79 5.813920000 -0.902296000 0.962772000

16 2.263750000 -1.779664000 -3.205028000

16 -1.122076000 -0.917789000 3.701225000

16 -1.213742000 -4.082869000 -0.727348000

16 -2.885280000 2.051472000 2.493058000

16 0.291046000 3.856484000 -0.957498000

16 2.478882000 -3.778151000 2.193837000

16 -0.324223000 1.859147000 -4.138656000

16 4.095705000 2.018573000 -2.424664000

16 1.689572000 3.183118000 3.068675000

16 -4.698185000 -2.869983000 1.337053000

16 -4.282281000 3.254226000 -2.057391000

16 6.445330000 -2.086301000 -1.003780000

16 5.012598000 -0.178732000 3.063970000

16 -4.837024000 -1.311839000 -3.109878000

6 4.498627000 3.848486000 -2.015436000

6 3.726661000 4.731188000 -3.048235000

6 7.812480000 -1.175277000 -1.972229000

6 7.738412000 -1.722185000 -3.432942000

6 3.689845000 -5.220662000 1.898634000

6 4.687706000 -5.148428000 3.102870000

6 6.309740000 0.899193000 3.962943000

6 6.307034000 2.332838000 3.359871000

6 1.927793000 -3.539646000 -3.880831000

6 0.565662000 -3.511538000 -4.642350000

6 -0.323719000 1.218586000 -5.937983000

6 -1.750723000 0.649129000 -6.226426000

6 -6.591424000 -1.496248000 -3.878570000

6 -6.953814000 -3.016170000 -3.838412000

6 -4.912933000 4.451115000 -3.387378000

6 -4.953256000 5.872394000 -2.709138000

6 -3.968064000 3.626201000 2.380915000

6 -5.205396000 3.227075000 1.517728000

6 -6.366430000 -2.877827000 2.247543000

6 -6.140768000 -3.836009000 3.462184000

6 0.323702000 5.175433000 0.340446000

1 1.382513000 5.356211000 0.545330000

1 -0.130568000 4.796604000 1.252970000

6 -0.376059000 6.470030000 -0.074558000

6 -0.562438000 -2.205998000 5.003506000

6 -1.283979000 -1.758896000 6.320595000

6 -2.503875000 -5.265047000 -0.095553000

1 -3.162759000 -4.742163000 0.593080000

1 -1.969919000 -6.032103000 0.464046000

6 -3.338399000 -5.936217000 -1.209767000

6 1.842449000 3.940898000 4.810640000

6 1.018384000 3.148187000 5.866056000

1 4.130631000 -5.434966000 4.003768000

1 5.314931000 2.759299000 3.545693000

1 6.765127000 -1.423696000 -3.841142000

1 -2.977423000 -6.952713000 -1.386968000

1 0.040338000 2.939331000 5.409748000

1 -4.836857000 2.960523000 0.524017000

1 -5.454355000 -3.331240000 4.152900000

1 -0.810425000 -0.823465000 6.644376000

1 -1.429398000 6.251786000 -0.282014000

1 -5.188187000 5.719685000 -1.647315000

1 -6.121876000 -3.570477000 -4.289933000

1 -2.467445000 1.463233000 -6.063954000

1 2.660749000 4.509875000 -2.925464000

1 0.736046000 -3.088553000 -5.635215000

6 5.841173000 -6.195584000 2.987617000

8 5.723239000 -7.297782000 2.500973000

8 6.985708000 -5.760667000 3.523208000

6 7.298457000 3.316839000 4.052169000

8 7.641127000 3.256407000 5.210637000

8 7.691782000 4.310402000 3.243467000

6 8.808034000 -1.033925000 -4.319852000

8 8.672848000 0.081096000 -4.769536000

8 9.898988000 -1.781006000 -4.527224000

6 -4.880349000 -6.003838000 -0.851819000

8 -5.624609000 -5.833324000 -1.867644000

8 -5.173537000 -6.199109000 0.334828000

6 1.566588000 1.755914000 6.250665000

8 1.137257000 1.348937000 7.451324000

8 2.263274000 1.057570000 5.545192000

6 -6.165800000 4.423366000 1.299124000

8 -5.975528000 5.300199000 0.478995000

8 -7.249765000 4.391343000 2.077185000

6 -7.456007000 -4.047252000 4.284881000

8 -7.684446000 -5.327135000 4.554188000

8 -8.171857000 -3.141381000 4.657938000

6 -1.055974000 -2.749801000 7.505627000

8 -2.073463000 -2.756263000 8.373719000

8 -0.060811000 -3.419163000 7.666279000

6 -0.388298000 7.433442000 1.145037000

8 -0.162923000 8.695757000 0.812810000

8 -0.614510000 7.057171000 2.281128000

6 -3.617540000 6.659206000 -2.718652000

8 -3.790310000 7.973891000 -2.775823000

8 -2.509907000 6.155904000 -2.638516000

6 -8.188833000 -3.301623000 -4.756323000

8 -8.138451000 -3.263804000 -5.964990000

8 -9.290280000 -3.593766000 -4.066588000

6 -1.895397000 0.263787000 -7.713780000

8 -1.723120000 -1.045885000 -7.988042000

8 -2.141949000 1.068780000 -8.577223000

6 3.882929000 6.236728000 -2.739768000

8 3.210184000 6.824383000 -1.916164000

8 4.830732000 6.838488000 -3.460727000

6 -0.088818000 -4.954284000 -4.822258000

8 -1.293754000 -4.991260000 -4.366454000

8 0.579288000 -5.829739000 -5.354266000

7 4.216769000 4.517989000 -4.421471000

1 5.131331000 6.153893000 -4.107942000

1 3.488844000 4.749022000 -5.093462000

7 -5.979700000 6.796426000 -3.223937000

1 -6.883002000 6.637714000 -2.791325000

1 -6.091084000 6.734129000 -4.232416000

7 -5.991174000 2.148537000 2.147957000

1 -7.189718000 3.523428000 2.550824000

1 -5.394882000 1.521010000 2.681264000

7 -5.594492000 -5.163544000 3.143878000

1 -6.918978000 -5.797114000 4.116272000

1 -4.612609000 -5.233536000 3.385150000

7 6.532023000 2.438846000 1.911085000

1 7.314364000 4.094660000 2.359549000

1 5.715079000 2.118360000 1.400928000

7 7.971722000 -3.175290000 -3.494675000

1 9.695041000 -2.656508000 -4.124022000

1 7.505516000 -3.575721000 -4.304534000

7 -3.308925000 -5.257754000 -2.549570000

1 -3.292638000 -4.241693000 -2.437932000

1 -4.282507000 -5.501458000 -2.882991000

7 -7.286670000 -3.512927000 -2.497977000

1 -6.970623000 -2.889754000 -1.761074000

1 -6.855905000 -4.423433000 -2.302729000

7 -2.055487000 -0.530078000 -5.390757000

1 -1.998639000 -0.239269000 -4.411231000

1 -1.587641000 -1.517181000 -7.147050000

7 -0.518266000 -2.705866000 -3.991110000

1 -0.256726000 -2.288374000 -3.093519000

1 -1.237169000 -3.500084000 -3.879561000

7 5.305163000 -3.845743000 3.388828000

1 5.539680000 -3.336795000 2.539292000

1 6.789828000 -4.832627000 3.808543000

1 7.606013000 -3.652353000 -2.674594000

1 4.693792000 -3.240987000 3.928426000

1 7.313998000 1.857591000 1.617278000

1 -4.774627000 8.086390000 -2.886987000

1 4.460203000 3.542836000 -4.578474000

1 -2.530121000 -5.502440000 -3.196660000

1 -0.908188000 -1.966484000 -4.599123000

1 -3.033706000 -0.793192000 -5.511840000

1 -6.442878000 1.581328000 1.435237000

1 -5.670346000 -5.433362000 2.159997000

1 -8.969533000 -3.658609000 -3.125795000

6 5.836560000 0.867431000 5.426533000

1 4.801979000 1.202077000 5.528448000

1 5.884942000 -0.156438000 5.809132000

1 6.486873000 1.505044000 6.027482000

6 7.679834000 0.221128000 3.837354000

1 7.635683000 -0.810093000 4.196296000

1 8.048651000 0.194861000 2.806422000

1 8.400068000 0.768443000 4.454321000

6 4.070791000 4.173673000 -0.583355000

1 2.999642000 4.033162000 -0.445592000

1 4.597931000 3.524484000 0.117650000

1 4.318875000 5.209761000 -0.336253000

6 6.024037000 3.984868000 -2.155328000

1 6.383523000 3.718959000 -3.151329000

1 6.322401000 5.018684000 -1.947416000

1 6.526972000 3.340892000 -1.429497000

6 9.135571000 -1.541940000 -1.278948000

1 9.138038000 -1.180330000 -0.247003000

1 9.315275000 -2.619185000 -1.261446000

1 9.969612000 -1.065744000 -1.805252000

6 7.581125000 0.336421000 -1.933330000

1 8.376808000 0.852312000 -2.476041000

1 6.627917000 0.612307000 -2.387612000

1 7.582881000 0.681174000 -0.894843000

6 2.857505000 -6.511022000 1.972121000

1 2.150603000 -6.557906000 1.138512000

1 3.532449000 -7.367977000 1.923626000

1 2.289665000 -6.565960000 2.906273000

6 4.399544000 -5.105424000 0.549633000

1 5.047653000 -5.975886000 0.408765000

1 3.670022000 -5.083089000 -0.262067000

1 5.011123000 -4.201918000 0.475649000

6 1.973415000 -4.581820000 -2.762510000

1 1.204035000 -4.419426000 -2.007121000

1 1.833643000 -5.577509000 -3.193191000

1 2.947589000 -4.545786000 -2.269902000

6 3.022599000 -3.838611000 -4.918910000

1 3.995018000 -3.941982000 -4.429550000

1 2.765726000 -4.778751000 -5.417246000

1 3.094645000 -3.047354000 -5.671372000

6 -6.351741000 3.979352000 -3.671809000

1 -6.812173000 4.588706000 -4.456066000

1 -6.339348000 2.945519000 -4.026800000

1 -6.981320000 4.027085000 -2.778348000

6 -4.076418000 4.404613000 -4.669207000

1 -3.024617000 4.616251000 -4.475479000

1 -4.158717000 3.420736000 -5.139706000

1 -4.452858000 5.145300000 -5.388323000

6 -4.356894000 3.963151000 3.831538000

1 -4.869159000 3.140473000 4.335639000

1 -3.464672000 4.217391000 4.409585000

1 -5.020718000 4.835221000 3.839994000

6 -3.202397000 4.794345000 1.763595000

1 -2.325411000 5.043689000 2.363962000

1 -2.876611000 4.560746000 0.746724000

1 -3.841102000 5.680302000 1.716485000

6 0.788283000 0.197429000 -6.192377000

1 0.724620000 -0.666125000 -5.531312000

1 0.753900000 -0.147439000 -7.232517000

1 1.764901000 0.656524000 -6.021393000

6 -0.079147000 2.483057000 -6.780759000

1 0.880495000 2.928669000 -6.505696000

1 -0.056118000 2.231846000 -7.844517000

1 -0.862067000 3.229724000 -6.627066000

6 -6.409830000 -0.985985000 -5.318170000

1 -5.707341000 -1.605274000 -5.882386000

1 -7.360972000 -1.017644000 -5.851816000

1 -6.047115000 0.046118000 -5.302557000

6 -7.624443000 -0.637844000 -3.138861000

1 -8.611014000 -0.787555000 -3.591865000

1 -7.700562000 -0.883186000 -2.077105000

1 -7.366730000 0.421470000 -3.218441000

6 -7.415682000 -3.446889000 1.281225000

1 -7.584839000 -2.754875000 0.451696000

1 -7.128252000 -4.416689000 0.866551000

1 -8.366058000 -3.555008000 1.814453000

6 -6.735942000 -1.457491000 2.684156000

1 -5.976425000 -1.040227000 3.352937000

1 -6.816398000 -0.813549000 1.802963000

1 -7.687676000 -1.472450000 3.217866000

7 0.732260000 3.855908000 7.128566000

1 1.561323000 4.315226000 7.499449000

1 0.685673000 2.138965000 7.839090000

1 0.018173000 4.565698000 7.004688000

7 -2.730432000 -1.490697000 6.248426000

1 -2.909134000 -0.587272000 5.821150000

1 -2.750902000 -2.155623000 7.981075000

6 -0.945181000 -3.629372000 4.588095000

1 -0.507320000 -3.880284000 3.619133000

1 -0.563269000 -4.332712000 5.334253000

1 -2.030028000 -3.760456000 4.506589000

6 0.954140000 -2.058121000 5.162073000

1 1.241429000 -1.035772000 5.416237000

1 1.284736000 -2.720250000 5.965276000

1 1.463522000 -2.339991000 4.240410000

1 -3.204440000 -2.183030000 5.672471000

6 3.326702000 4.047614000 5.174901000

1 3.444967000 4.557069000 6.139906000

1 3.796148000 3.067743000 5.249408000

1 3.859583000 4.649513000 4.433202000

6 1.245815000 5.351336000 4.644626000

1 0.183045000 5.328150000 4.389042000

1 1.372637000 5.930388000 5.566258000

1 1.753496000 5.892180000 3.843893000

7 0.157920000 7.183189000 -1.235894000

1 0.017224000 8.649336000 -0.166426000

1 -0.409693000 7.002731000 -2.058944000

1 1.137624000 6.976731000 -1.427173000
